# Intravenous misplacement of the nephrostomy catheter following percutaneous nephrostolithotomy: two case reports and literature review

**DOI:** 10.1186/s12894-017-0233-3

**Published:** 2017-06-14

**Authors:** Weijin Fu, Zhanbin Yang, Zhibin Xie, Haibiao Yan

**Affiliations:** Department of Urology, The First Affiliated Hospital of GuangXi Medical University, 6 Shuangyong Road, Nanning, 530021 GuangXi Zhuang Autonomous Region People’s Republic of China

## Abstract

**Background:**

Intravenous misplacement of a nephrostomy tube after percutaneous nephrostolithotomy (PCNL) is very rare in clinical experiences. This report summarizes the characteristics and management of intravenous misplacement.

**Case presentation:**

We present two uncommon cases of intravenous nephrostomy catheter misplacement after PCNL from among 4220 patients who underwent PCNL between January 2009 and December 2015. The tip of the tube was located in the inferior vena cava in one case and in the renal vein in the other. We preferably performed open surgery to treat the two patients, mainly to remove the residual calculi and to prepare for any possible adverse event. All patients were successfully managed and discharged uneventfully.

**Conclusion:**

Intravenous nephrostomy tube misplacement is an uncommon PCNL complication. Furthermore, the study illustrates the importance of prompt diagnosis of renal vein perforation and its prompt management using open surgery, similar to conservative therapies.

## Background

Percutaneous nephrostolithotomy (PCNL) is a minimally invasive procedure to remove kidney stones more than 2 cm in size. A nephrostomy catheter is routinely retained in the renal pelvis to compress bleeding and drain the fluid and urine from the collecting system after surgery. Although PCNL is generally safe and effective, it is occasionally associated with uncommon complications [1]. Intravenous misplacement of a urological catheter is an uncommon complication associated with PCNL.

Therefore, the mechanism and proper management of the uncommon complication should be investigated. However, few publications have reported intravenous nephrostomy tube misplacement. We researched all articles listed during the last 15 years(between January 2002 and December 2016) in the PubMed database. The search procedure was performed to identify all relevant trials retrieved using the following search terms: “intravenous misplacement or nephrostomy tube misplacement or misplacement”, and “percutaneous nephrostolithotomy(PCNL)”, and “inferior vena cava or vena cava or renal vein”, and “Foley catheter or nephrostomy tube”,with the last term being the most important.

We have retrospectively evaluated the clinical data of 4220 patients who underwent PCNL in a single institution between January 2009 and December 2015.Among the patients, 2546 were male (60.3%) and 1674 female (39.7%).Single calculi were treated in 1987 cases, there were 1012 patients of multi calculi, and 1221 patients of upper ureteral calculi. 2490 patients were treated in prone position, 1730 patients in lateral position. 2380 patients underwent by fluoroscopy-guided, 1840 patients by ultrasound-guided. 60 patients(1.4%) converted to open surgery, and 6 patients lost the diseased kidney due to refractory bleeding in the early stage. 100 (2.3%) patients received blood transfusions and 30 (0.7%) patients needed highly selective renal artery embolization.

Intravenous nephrostomy tube misplacement after PCNL occurred in 1 of 4220 patients on February,2014 during mature technology phase. Another patient with intravenous misplacement of a nephrostomy tube, who underwent PCNL in another hospital, was transferred to our hospital. We have summarized our experiences with these two uncommon cases.

## Case presentation

### Case 1

A 68-year-old male patient underwent PCNL for a staghorn calculus in the right kidney. The PCNL was performed in the prone position. The puncture site was localized to the 11th intercostal space between the posterior axillary line and scapular line. **F**luoroscopy-guided percutaneous punctures were performed with an 18-gauge needle by retrograde pyelography. A zebra guide wire was inserted into the collecting system. Access to the excretory system was achieved gradually by fascial dilators. Immediately after dilator removal, severe bleeding from the sheath led to a sudden interruption of the procedure; an 18 F nephrostomy catheter was promptly inserted and closed to control the bleeding. The blood loss was estimated 500 ml.The blood pressure had dropped. After blood transfusion, hemodynamics returned to normal.

**Fig. 1:**
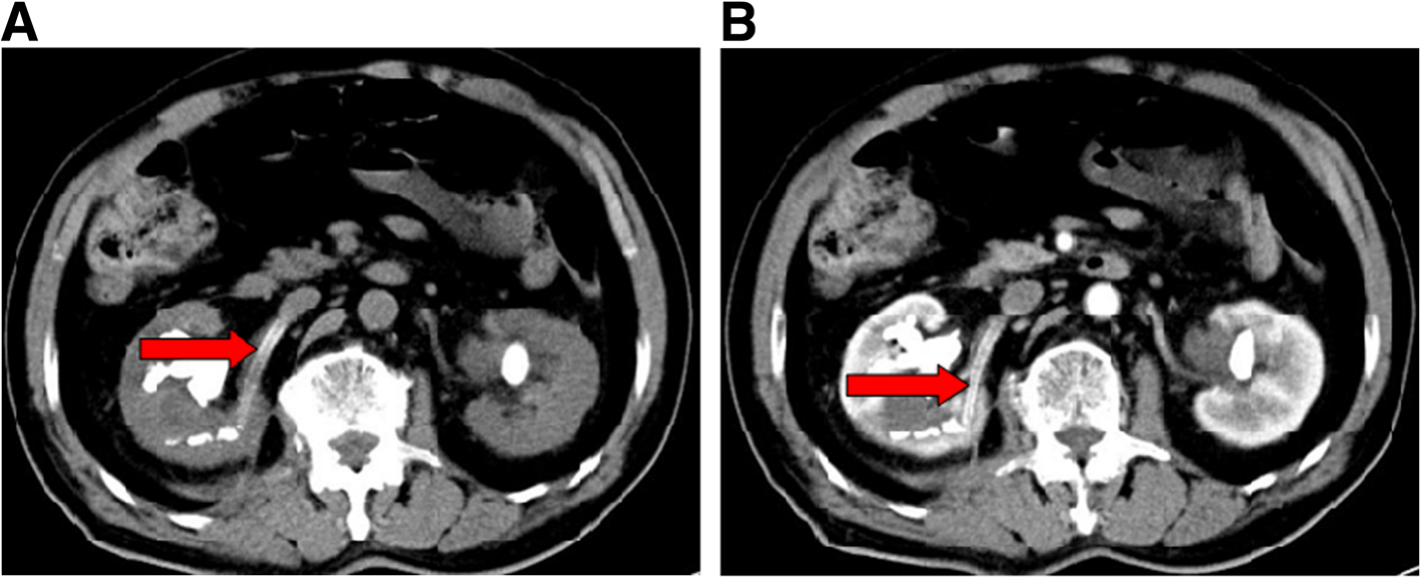
CT revealed the nephrostomy tube piercing into the right renal vein. **a** Plain of CT demonstrated nephrostomy catheter (*red arrow*) had traversed the lower pole of right kidney directly into the *right* renal vein. **b** Enhanced CT scan revealed that nephrostomy catheter (*red arrow*) had transversed *left* renal vein

Enhanced computed tomography scan, performed 2 days after the surgery showed that the nephrostomy catheter had traversed the lower pole of the right kidney directly into the right renal vein **(**Fig. 1 **)**. Exploratory laparotomy was performed under general anesthesia, in the event of massive bleeding, on the 7th postoperative day. During the operation, the appearance of renal vein was normal. No bleeding of kidney or renal vein occurred after removing the nephrostomy catheter. Simultaneously, the staghorn calculus of the right kidney was removed via anatrophic nephrolithotomy, and a double J stent was indwelled. The patient was discharged uneventfully on the 14th postoperative day.

### Case 2

**Fig. 2:**
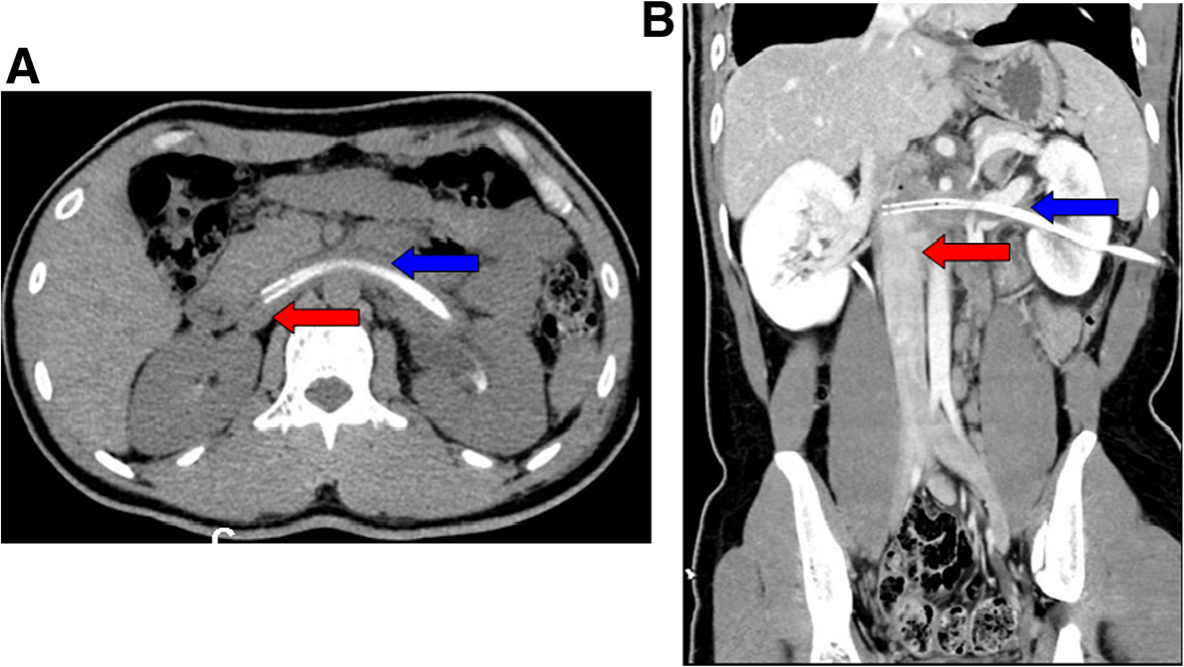
CT revealed the nephrostomy tube piercing into IVC. **a** Plain of CT scan revealed that nephrostomy catheter (*blue arrow*) had transversed *left* renal vein directly into IVC (*red arrow*). **b** Enhanced CT scan demonstrated that nephrostomy catheter (*blue arrow*) had transversed *left* renal vein directly into IVC (*red arrow*)

A 28-year-old male patient underwent ultrasound-guided PCNL for left upper ureteral calculi, which was 1.2 cm in size, in another hospital. The procudure was performed in the prone position. Access to the excretory system was achieved by fascial dilators. A zebra guide wire was retained during the procedure. Severe venous bleeding was noted during the dilating process. The procedure was interrupted, and a nephrostomy catheter was inserted and closed to control bleeding. The nephrostomy catheter was reopened on the 7th postoperative day, and severe bleeding was observed through the drainage catheter, which was immediately closed. Subsequently, computed tomographic angiography (CTA) showed that the nephrostomy catheter had transversed the left renal parenchyma, misplaced from the left renal vein, directly into the inferior vena cava (IVC) **(**Fig. 2 **)**.

The patient was urgently transferred to our institution. Before we did the exploration, we consulted with vascular surgery. The vascular surgery team encouraged us to proceed safely, confirming that there would be no bleeding. Simultaneously they advised to begin anticoagulant therapy and monitoring. In the end, the patient was done as planned.

Exploratory laparotomy was performed under general anesthesia on the 14th postoperative day. Although the nephrostomy catheter was removed, a small amount of blood oozed from the rupture of the left kidney, and the patient remained hemodynamically stable. A 2-0 Vicryl suture was used to stitch up the ruptured left renal parenchyma. Simultaneously, the left upper ureteral calculus was removed by ureterolithotomy. A 4-0 Vicryl suture was used to interrupted suture the ureteral incision and a double J stent was indwelled. The patient was discharged uneventfully on the 21st postoperative day.

## Discussion

Although PCNL is an established procedure, it has been associated with some complications, including hemorrhage, sepsis, kidney or adjacent organ (such as, the liver, spleen, and bowel) injury, access lost, excretory system perforation, and so on. Hemorrhage is the most significant PCNL complication. Venous bleeding during percutaneous procedures is mild and it ceases spontaneously or detains the nephrostomy catheter into the renal pelvis [2]. Severe bleeding complications of PCNL are mainly associated with arterial injuries [3].

Placing the nephrostomy catheter into the collecting system is an effective method for compressing venous bleeding. The catheter can occasionally pierce the renal parenchyma and migrate into the renal vein and even the IVC. Several publications have presented the rare complications with nephrostomy catheter misplacement into the vessel after PCNL [3–9] in the PubMed database. The data from these publications are summarized in Table 1.Similar to the reports of other centers, the incidence of intravenous nephrostomy tube misplacement after PCNL was 0.23% (1/4220) at our institutions.

**Table 1:** Reports of intravenous misplacement of a nephrostomy tube

Author	No./sex/age	Side	Catheter	Catheter withdrawl	Operation type	Location	Subsequent treatment
Dias Filho, et al.	1/F/63	L	Foley catheter	1-step under fluoroscopy	Catheter placement	Renal vein, IVC	Second PCNL
Shaw G, et al.	2/M54	R	Nephrostomy tube	2-step under fluoroscopy	PCNL	Renal vein	Exploratory
Mazzucchi E, et al.	3/M/52	L	Nephrostomy tube	1-step under fluoroscopy	PCNL	Renal vein	No
Mazzucchi E, et al.	4/F/35	L	Nephrostomy tube	2-step under fluoroscopy	PCNL	Renal vein, IVC	No
Li, et al.	5/F/32	L	Nephrostomy tube	2-step under fluoroscopy	PCNL	Renal vein, IVC	No
CJ,Wang, et al.	6/F/66	L	Nephrostomy tube	1-step under fluoroscopy	PCNL	Renal vein	No
Kotb, et al.	7/M/50	L	Foley catheter	1-step open pyelotomy	Catheter placement	Renal vein, IVC	Open pyelotomy
XF Chen, et al.	8/M/42	L	Nephrostomy tube	2-step under CT guide	PCNL	Renal vein, IVC	Second PCNL
XF Chen, et al.	9/F/38	L	Nephrostomy tube	2-step under fluoroscopy	PCNL	Renal vein, IVC	Simultanuous PCNL
XF Chen, et al.	10/M/48	L	Nephrostomy tube	1-step under ultrasound	PCNL	Renal vein	Second PCNL

*PCNL* percutaneous nephrostolithotomy, *M* male, *F* female, *L* left, *R* right, *IVC* inferior vena cava

The rare complications have been attributed to the following causes. First, the dilator sheath has likely penetrated the renal parenchyma and directly injured the renal vein. Subsequently, perforation into the renal vein by the zebra guide wire, with dilatation of the injured vein, resulted in the nephrostomy catheter migrating to the venous system. Second, the rupture in a large renal vein branch caused by the instruments used during intervention was the most likely cause of the observed bleeding. To control bleeding, the nephrostomy catheter was inadvertently inserted into the venous system, even the IVC. The two cases contributed to the former.

Based on previous articles, hemorrhage control could be achieved with a nephrostomy catheter, despite perforation into the major renal vein. The tract was allowed to heal with the catheter being withdrawn in stages, under fluoroscopic guidance. Similar to the other reported cases, Xiao-Feng Chen and colleagues [8] reported three cases of intravenous misplacement after PCNL. The tip of the tube was located in the IVC in two cases, and in the renal vein in one case. All cases were successfully managed with one-step (one case) or two-step (two cases) tube withdrawal, while under close monitoring.

Unlike conservative therapies, we had preferably performed open surgery under general anesthesia in two patients, mainly to remove the right renal calculi in one case and the left ureteral calculi in the other, and to be prepared for any possible adverse events. If the two patients had no residual stone, the misplaced nephrostomy tube can be successfully removed through strict bed rest, intravenous antibiotics, and under close monitoring.

## Conclusions

Intravenous misplacement of a nephrostomy tube is an uncommon complication after PCNL. Although PCNL and nephrostomy catheter exchange are relatively simple procedures, they should be cautiously performed preferably under ultrasound or fluoroscopic guidance. Combined with literature, most patients may be managed conservatively with strict bed rest, intravenous antibiotics, and tube withdrawal by CT or fluoroscopy guide. Open surgery can be used as an alternative treatment.

## Abbreviations

CT: Computed tomography
CTA: Computed tomographic angiography
IVC: Inferior vena cava
PCNL: Percutaneous nephrostolithotomy.
